# Neurodynamical Computing at the Information Boundaries of Intelligent Systems

**DOI:** 10.1007/s12559-022-10081-9

**Published:** 2022-12-27

**Authors:** Joseph D. Monaco, Grace M. Hwang

**Affiliations:** 1 Dept of Biomedical Engineering, Johns Hopkins University School of Medicine, Baltimore, MD USA; 2 Johns Hopkins University Applied Physics Laboratory, Laurel, MD USA

**Keywords:** Artificial intelligence, Embodied cognition, Perceptual control theory, Dynamical systems, Computational neuroscience, Robotics

## Abstract

Artificial intelligence has not achieved defining features of biological intelligence despite models boasting more parameters than neurons in the human brain. In this perspective article, we synthesize historical approaches to understanding intelligent systems and argue that methodological and epistemic biases in these fields can be resolved by shifting away from cognitivist brain-as-computer theories and recognizing that brains exist within large, interdependent living systems. Integrating the dynamical systems view of cognition with the massive distributed feedback of perceptual control theory highlights a theoretical gap in our understanding of nonreductive neural mechanisms. Cell assemblies—properly conceived as reentrant dynamical flows and not merely as identified groups of neurons—may fill that gap by providing a minimal supraneuronal level of organization that establishes a neurodynamical base layer for computation. By considering information streams from physical embodiment and situational embedding, we discuss this computational base layer in terms of conserved oscillatory and structural properties of cortical-hippocampal networks. Our synthesis of embodied cognition, based in dynamical systems and perceptual control, aims to bypass the neurosymbolic stalemates that have arisen in artificial intelligence, cognitive science, and computational neuroscience.

## Main

The current paradigm of artificial intelligence and machine learning (AI/ML) casts intelligence as a problem of representation learning and weak compute. This view might be summarized as the hope that defining features of biological intelligence will be inevitably unlocked by learning network architectures with the right connectional parameters at a sufficient scale of compute (i.e., proportional to input, model, and training sizes) to be attained in the future. Despite increasingly impressive capabilities, the latest and largest models, like GPT-3, OPT-175, DALL-E 2, and a recent “generalist” model called Gato [1–6], continue a long history of moving the goalposts for these biological features [7–11], which include abstraction and generalization; systematic compositionality of semantic relations; continual learning and causal inference; as well as the persistent orders-of-magnitude performance gaps reflected by low sample-complexity and high energy-efficiency. Instead of presuming that scaling beyond trillion parameter models will yield intelligent machines from data soup, we infer that understanding biological intelligence sufficiently to formalize its defining features likely depends on characterizing fundamental mechanisms of brain computation and, importantly, solving how animals use their brains to construct subjective meaning from information [12–19]. In this paper, we first interpret the disciplinary history of approaches to these questions to illuminate underexplored hypothesis spaces. Second, we motivate a perceptual control-theoretic perspective and discuss implications for computing in physical dynamical systems. Third, we synthesize properties of mammalian cortical oscillations and structural connectivity to argue for a nonreductive base layer, i.e., a supraneuronal mechanistic substrate, for neurocognitive computing in brains. To conclude, we discuss implications for bridging AI/ML gaps and incentivizing increased cooperation between theoretical and computational neuroscience, autonomous robotics, and efforts to develop brain-like computing models for AI.

## Framing an Integrative Neuroscience of Intelligence

To find space for integrative research in the neuroscience of intelligence, we will sketch the historical entanglement of several fields that have addressed neural computation and intelligence. First, current AI/ML research questions and methods range from reinforcement learning and multilayer neural nets to more recent transformers and graph diffusion models. These models, like those mentioned above, can learn impressive solutions in narrow domains like rule-based games and simulations or classification and recognition tasks. However, these domains substantially constrain the nonstationarity and dimensionality of problem sets, to the degree that merely emulating the intelligence reflected by massive amounts of a contextual training data can often yield effective problem-solving. Given the explosive growth, commercial success, and technological entrenchment of AI/ML, researchers and practitioners have, perhaps sensibly, prioritized the short-term reliable incrementalism of scale [20–22] over the uncertain and open-ended project of explicating and formalizing biological intelligence. It has been unclear how to encourage sustained consideration of biological insights in the field [23–25], especially given the prevalent belief that some combination of current approaches will eventually achieve artificial general intelligence, however defined.

Second, the “cognitive revolution” of the 1950s [26] installed blinders on both cognitive science and AI in their formative years. Reacting to behaviorism’s near total elision of the mind, the advent of computational cognitivism and connectionist neural nets [27–31] conversely stripped humans and other intelligent animals of everything else that adaptively embeds an organism in the world [32–36]. This rejection of agency, embodiment, interaction, homeostasis, temporality, subjectivity, teleology, etc., served to modularize and centralize minds within brains using superficial and incoherent brain–computer metaphors [37–40]. An overriding focus on the brain as the hardware of a computational mind, thought to fully encapsulate intelligence, may have contributed to the failure of the original computationalist branch of cognitive science to coalesce an interdisciplinary research program as first envisioned [26, 41]. To add to the collateral damage, the same blinders have skewed the conceptual language and methodologies of neuroscience [42–45] as it developed into a similarly sprawling field.

Third, computational neuroscience, originally a theoretical and analytical blend of physics, dynamical systems, and connectionism [46–53], found a path toward a working partnership with experimental neuroscience [54–58] largely by conforming to the latter’s neurocentric reductionism, exemplified by the 150-year dominance of the neuron doctrine [59, 60]. The most successful form of reductive modeling in neuroscience has typically targeted deductive, data-driven prediction and validation of experimental observations of single-neuron behavior. This biophysical approach is undergirded by the Hodgkin-Huxley formalism [61–63] which quantifies a sufficient subset of lower level, physical/chemical processes—e.g., ionic gradients, reversal potentials, intrinsic conductances, (in)activation nonlinearities, etc.—that remain analytically coherent at the subneuronal level of excitable membranes and cellular compartments. In contrast, modeling supraneuronal group behavior has relied on large-scale simulations of simple interconnected units, efficiently implemented as 1- or 2-dimensional point-neuron models (cf. [64]). These models are akin to connectionist nets that optionally feature computationally tractable features [65–72] including spiking units, lateral inhibition, sparse or recurrent connectivity, and plasticity mechanisms based on local synaptic modification rules (in contrast to the global weight-vector updates from the error backpropagation algorithms used to train AI/ML models). The field has yet to unlock network-level or macroscale approaches that match the explanatory or quantitative success of biophysical modeling.

This historical framing suggests that the multidisciplinary triangle of AI, cognitive science, and computational neuroscience has developed mutually interlocking biases, respectively, technology-driven incrementalism, neurocentric cognitivism, and synaptocentric emergentism. The synaptic learning bias has hindered computational neuroscience explanations for cognitive processes at scale, e.g., interregional cortical gating for learning sensory-cued behaviors [73] or embodiment-related questions of brain–body coupling and environmental interactions [34]. A scientific problem-solving culture that encouraged thinking at organismal—even social and societal—scales could have embraced theory-building as an essential, transformative practice [56]. However, on the road from cybernetics to reductive analysis tool, computational neuroscience bypassed systems-level modeling and inductive theory-building, thus shunting the field from the kind of scale that might have complemented AI. We argue below that new integrative approaches could cooperatively break the mutual frustrations of these three fields.

## Countering Observer Bias with Embodied Dynamical Control

A common source of the biases described above is the external observer perspective typically assumed in experimental neuroscience [74–76], i.e., that experimenters conceptualize their subjects as input-output systems in which the output is identified with behavior. Inverting this perspective entails seeing peripheral sensory transduction and motoneuron firing as mutual and simultaneous causes and effects of cognition and behavior. Paraphrasing Skarda (1999) [77], embodiment-first theories invert our view of cognition as integrating isolated channels of sensory information into unified internal models, to one of articulating dynamical boundaries within existing global states that already reflect an organism’s cumulative experience in its world (*umvelt*). In humans, this lifelong context of prior articulations inescapably conditions the meaning that we discern from the momentary flow of experience and, therefore, also guides our planning and future behavior. While Skarda’s argument is strictly noncognitivist and nonrepresentational, we consider these neurodynamical insights more broadly below.

A related position, the dynamical systems view of cognition, was presented by van Gelder (1995) as a viable alternative to computational metaphors [78–80]. This argument centralized the continuous temporality, i.e., rate, rhythm, timing, and coordination, of neural activity as causal interactions mutually unfold between an organism and its environment. A key consequence of temporality—and of the ability to express dynamical systems as differential equations—is to view cognitive systems as controllers, because timeliness of the response to a perturbation is a precondition for effective control [81–83]. Thus, in the dynamical view, the organization of brain states by time, and not merely by sequential ordering, supports an understanding of cognition as a fundamentally dynamical (noncomputational) controller. van Gelder anchored this argument with a case study of Watt’s eighteenth-century invention of the steam governor [78], which, while compelling, revealed some limitations. In particular, the governor is indeed a continuous-time dynamical controller, but its sole function follows from its design which is necessarily entangled with the intentions, goals, and various capabilities of James Watt as he developed it. Its apparent intelligence is not its own but that of a high-embodiment human engineer; the governor is a tool, not a cognitive agent. More generally, attributing intelligence to low-embodiment tools, machines, or AI/ML models recapitulates the same category error. A related epistemic danger, particularly for high-dimensional artifacts like large language models or generative text-to-image networks, is that increasingly interesting control failures can be mistaken for the emergent complexity of intelligent behavior per se. We next elaborate a control-theoretic interpretation to clarify these distinctions.

## Perceptual Control of Self vs. Nonself Entropy

The dynamical view makes a positive case for continuous temporality and control, but the controller for a cognitive agent must be internally referenced, i.e., the command signals that are compared to inputs to produce feedback are set by the agent. Stipulating goal-setting autonomy simply recognizes the agency inherent in embodied life: animals have goals and those goals govern their behavior. Accordingly, Powers (1973) [84] introduced perceptual control theory as a hierarchically goal-driven process in which closed-loop negative feedback reduces deviations of a perceived quantity from an internal reference value; this formulation of biological control was overshadowed by the rise of cybernetics and its long influence over AI, cognitive science, and robotics. Renewed interest in perceptual control reflects recent arguments that the forward comparator models typical of cybernetics research locked in confusions about fundamental control principles [81–83], which may have subsequently influenced conceptual developments in neuroscience.

Under perceptual control, behavior is not itself controlled; instead, behavior is the observable net result of output functions that express sufficient motor variability, given simultaneous unobserved or unexpected forces acting on the body and environment, to satisfy an organism’s drives and achieve its goals [81–87]. Perceptual control shares this view of behavior with an alternative theoretical framework known as *radical predictive processing* [35, 88]. In this framework, the normative minimization of global prediction errors (viz. variational free-energy [89]) yields a distributed generative model that naturally encompasses an extended brain–body system [90]. Probabilistic Bayesian inference of perceptual hypotheses produced by this generative model arises through *active inference* [91, 92], i.e., actions and perceptions that maximize model evidence by balancing internal active-state (self) entropy with external sensory-state (nonself) entropy. Active inference thus reflects a compelling insight: agents learn distributed feedback models by adaptively balancing information streams along the self–nonself boundary. More generally, predictive processing assumes that living systems are autopoietic, entailing that system states follow ergodic trajectories. However, we do not find ergodicity to be obviously compatible with the distributional changes in organization that occur across an organism’s lifetime. Thus, we pursue perceptual control here, but with this additional insight that intelligence requires adaptively balancing self vs. nonself entropy.

By taking a perceptual control view, behavior becomes the lowest-level output of the reference signals descending through hierarchically nested controllers, while higher-level outputs encapsulate the externally unobservable processes of cognition, perception, and other aspects of intelligence. Internally referenced control of perceived inputs means that the feedback functions operate in closed loops through the body and the environment. The functional payoff is that an agent’s controller system has access to all of the information it needs to learn what it needs to learn at any moment. Conversely, there is no need for the more complex, error-prone process of integrating sensory data into internal representations and forward models of body kinematics, physics, planning, etc., to predict which motor outputs will achieve a given behavioral goal in an unfamiliar situation. In perceptual control, ascending inputs flow via comparator functions as deviations to the level above, and descending references flow via output functions as error-correcting signals to the level below. Additionally, we can broadly divide a cognitive agent’s inputs into two categories: internal embodied (interoceptive, homeostatic) signals and external situated (exteroceptive, allostatic) signals. Thus, by closing the hierarchical control loop with rich, continuous, organismal feedback, the problem of intelligent behavior shifts from representational model-building to the adaptive management of ascending/descending and self/nonself information streams. We can see now that the steam governor—with mechanical linkages instead of adaptively balanced information boundaries—is not the kind of device that can do the work of intelligence. We next consider the temporal continuity of dynamical systems.

## Do Brains Compute with Continuous Dynamics?

The dynamical systems view gives us mutual causal coupling and the unfolding temporality of neural states, but its formalization in differential equations implies that state trajectories are continuous in time. We discuss several implications of this constraint. First, the nature of computing with continuous functions is unclear; e.g., logical operations like checking equality of two real values or conditional execution (“if *x* > 0, do this; else, do that”) require discrete state changes that violate local continuity. To formalize a theory of analog computing, Siegelmann and Fishman (1998) resolved this difficulty by explicitly disallowing those operations internally but also stipulating that input and output units be discrete [93]. Further analyses of analog neural nets with continuous-valued units and weighted linear inputs revealed impressive capacities for “hypercomputational” transformations of high-dimensional vector spaces [94, 95]. Thus, despite continuous-time recurrent dynamics like neural circuits, the capabilities of analog neural nets mirrored both the generative power and logical-symbolic gaps of AI/ML models. Although structurally and operationally distinct, these two “neural” paradigms share the external-observer bias that mistakes cognitive computing as a linear series of input-output transformations. Dynamical complexity itself is not enough.

Second, the mere fact of neuronal spiking, viz*.* highly stereotyped all-or-none communication pulses, convinced McCulloch, Pitts, and von Neumann in the formative years of computer technology and symbolist AI that neurons were binary logical devices [96, 97]. While the idea proved useful for computer engineering, the biology of it was wrong enough for Walter Freeman (1995) to call it a “basic misunderstanding of brain function” [98]. Connectionists gave up McCulloch and Pitts’ logical units for rectified linear (or similar) activation functions, yet rectification entails that a coarse-grained binary division still persists in the codebook, between those units with zero vs. nonzero activations for a given input. In sum, continuous-time dynamical systems hinder the formation of discrete states, and continuous-valued AI/ML neural nets are built atop degenerate subdigital states.

Third, and relatedly, prospects for modeling continuous-time, continuous-valued neural systems with emergent logical-symbolic operations remain unclear, bringing us back to the central “paradox” [29] that historically divided symbolists and connectionists [7–9]: how can a physical system learn and implement symbolic rules? The argument has more recently taken the form of whether—or not [10–12]—the impressive progress represented by large transformer models already reflects an unrecognized emergence of limited symbolic (e.g., compositional) capacities [3, 6, 99, 100]. Alternatively, theory-driven and simulation-based approaches [101] have shown that specially devised nonsymbolic neural nets can facilitate the developmental emergence of finite automata or Turing machine-like computing capacities [102–104]. However, rationalist considerations of the neural circuit regularization imposed by the “genomic bottleneck” [105]—bolstered by recent demonstrations of architectural chromatin changes to the genome and epigenome from early learning experiences in mice [106]—suggest that incremental synaptic modification is insufficient to explain the innate robustness and adaptability of simple neural circuits [38, 107]. Thus, to bridge this neurosymbolic divide, we seek a middle way that integrates embodiment-first cognition, perceptual control, and collective neurodynamics.

Lastly, if brains are responsible for determining the various segments, boundaries, and discriminations required for episodic memory, categorical abstraction, perceptual inference, etc., then cognition ought to be characteristically discrete [108]. Convergent lines of evidence, from mice to humans, support an abundance of spatiotemporally discrete neurocognitive states [109–115], including a characteristic duration—a third of a second—for embodiment-related states revealed by deictic primitives of human movement patterns [116, 117]. Thus, if a useful formalization of biological intelligence is possible, it will require an understanding of discrete neurocognitive states as computational states [118–120] and that understanding should reconcile uncertainties about the nature of computing in nested, distributed, physical dynamical systems like brains. We next elaborate what it means to consider a nonreductive mechanistic account of neural computing; then we discuss oscillatory and structural factors that may support mechanisms of discrete neurodynamical computing for embodied perceptual control.

## Toward a Nonreductive, Mechanistic Base Layer of Computation

Miłkowski (2013) established epistemological criteria for mechanistic accounts of neural computation in part by requiring a complete causal description of the neural phenomena that give rise to the relevant computational functions [121]. The causal capacities of that computational substrate are then necessarily grounded in constitutive mechanisms—i.e., causal interactions between subcomponents—at some level of organization of the physical-material structure of the brain. Reductionist accounts of neural computation posit that such constitutive mechanisms interlink every level of organization, from the macroscale to neurons to biophysics: one simply attributes computation to all material processes of the brain down to its last electron. Given the dire implications for understanding high-level cognition reductively, the search for an alternative mechanistic account becomes attractive.

What does a nonreductive hypothesis of computing in the brain look like? Instead of assuming a series of causal linkages from macroscale phenomena all the way down to the quantum realm, we presume that there is some middle level beyond which the net causal interactions among lower level components no longer influence the functional states and state transitions that constitute the computational system [121–123]. We call this level the base layer of neural computation by analogy to the physical layer of modern computer chips, which consists of the arrays of billions of silicon-etched transistors and conductors whose electronic states are identified with and identical to the lowest-level computational states of the device [124]. The constitutive mechanisms within the base layer itself provide all of the causal interactions necessary to process a given computational state into the next. Thus, a nonreductive base layer is a substrate that computes state trajectories and insulates that computation from the causal influences of lower-level phenomena.

Which level of organization might serve as a base layer for the high-level cortical computations supporting cognition, perception, and other aspects of biological intelligence? Following our discussions above, we consider several criteria for this base layer: the computing layer must (1) encompass a macroscale, hierarchical control structure over which it implements comparator, error, and output functions; (2) adaptively control access to internal and external information flows generated by physical embodiment and situated embedding in a causal environment; and (3) support discrete neurodynamical states and adaptive high-dimensional state transitions across timescales of neural circuit feedback (10 ms), conscious access (40–50 ms [125]), deictic primitives of embodied cognition (300 ms [116]), and deliberative cognitive effort (>1–2 s). In an earlier paper that otherwise correctly focused on the need to close feedback loops across levels, Bell (1999) considered the computing layer question and dismissed its attribution to the level of neuron groups by suggesting that group coordination mainly serves to average away the noise of single-neuron spike trains [126]; as a consequence, Bell argued for a reductionist account, including its requisite quantum implications. Detracting groupwise neuronal coordination as merely signal-to-noise enhancement reflects an impoverished, yet prevalent, view of what neurons and brains do.

## What is the Structural Connectivity Basis of Intelligence?

**Fig. 1:**
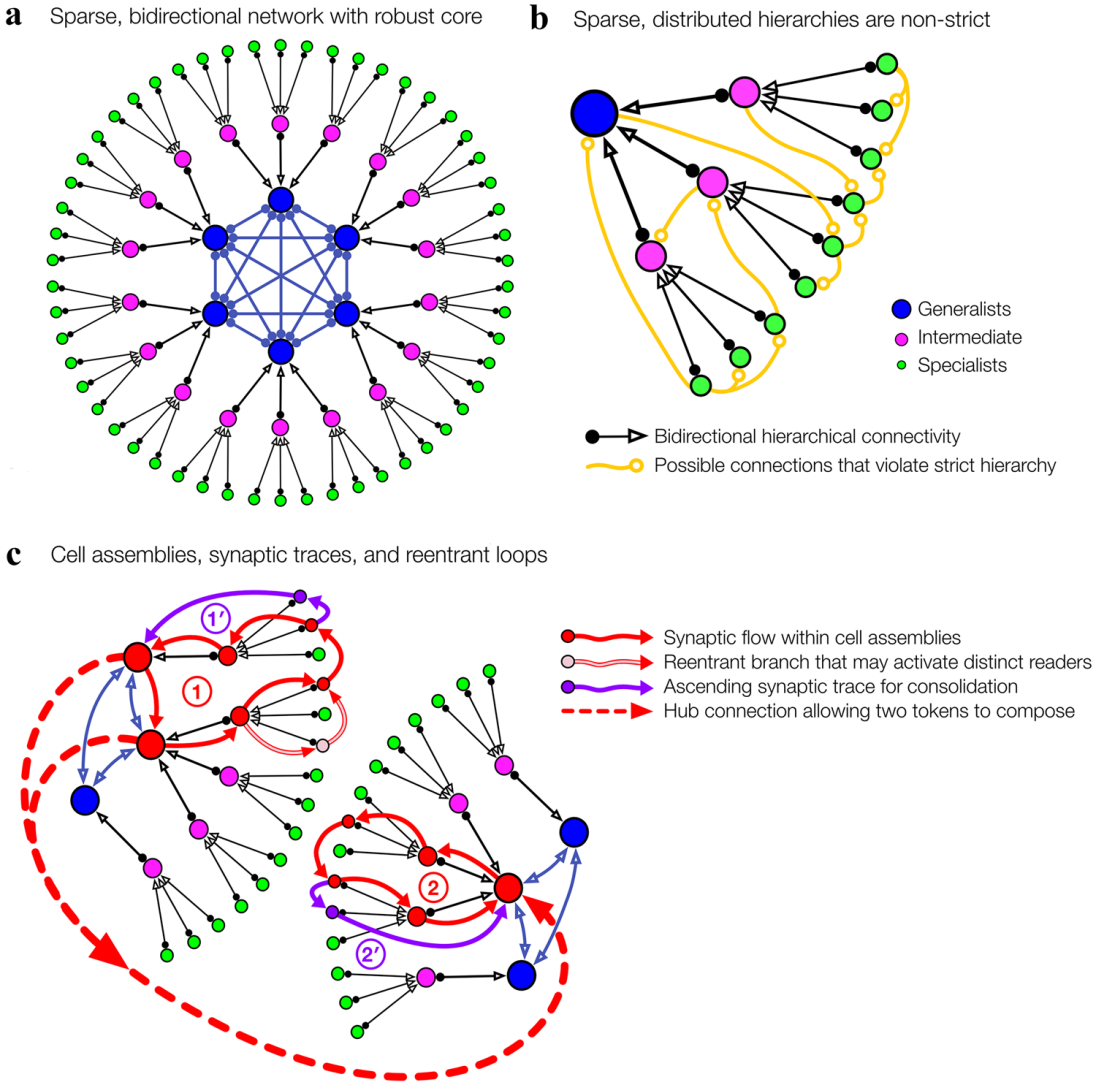
Brain networks form sparse heterarchies with log-skewed afferent connectivity.** a** A balanced hierarchy with a core subset of strongly interconnected generalist neurons, viz*.* a rich club. **b** Hierarchies strictly require binary, unambiguous superiority relations. However, neurons and neuronal networks inhabit a continuum of input connectivity from specialists to generalists. Sparse, multi-scale arrangements of elements from this skewed distribution naturally form heterarchies with lateral (e.g., modularity break) and ascending/descending (e.g., level skip) violations. **c** The adapting cell-assembly hypothesis implies that computation at the interface of information streams emanates from self-sustaining activations within the reentrant synaptic loops of interconnected cell assemblies (red circles, 1 and 2). Loops may branch into subloops or aggregate new traces (purple circles, 1′ and 2′) that entail different effects on downstream targets, thus instantiating new and distinct assemblies

Studies of cortical and hippocampal networks have revealed log-skewed distributions of mostly “specialist” neurons—with relatively few afferent inputs—and long tails of more excitable “generalist” neurons [74, 127]. The generalist neurons and networks can organize, despite their smaller numbers, into highly stable “rich clubs” [128–131] that robustly distribute higher levels of the hierarchy (Fig. 1a). Conceptual knowledge and remote memories are more robustly accessible than their specific and recent counterparts, due to more extensive consolidation of synaptic traces from hippocampal circuits to the medial temporal lobe and frontal areas [132–134]. Collectively, these properties suggest that the cortical-hippocampal memory graph is not a strict hierarchy, but more like a distributed, multilevel arrangement of specialist to generalist neurons. By including loops that skip levels or break modularity, brain networks are more appropriately considered heterarchies (Fig. 1b; cf. [135]). Sparse, log-skewed, heterarchical networks can feature numerous, diverse, connected subgraphs that may support latent cell assemblies.

In a recent study, optogenetic stimulation induced new place fields and revealed that memory formation is constrained to preconfigured network relationships inferred prior to stimulation [136]. Mature brains provide a vast library of arbitrary, yet stable, latent structures that can be recruited as needed. In contrast, synapses are highly variable due to fluctuations in transmission failure rates, dendritic spine turnover, and synaptic volume and strength [137, 138]. Thus, our hypothesis of supraneuronal computation is grounded in the relative structural stability of neuronal groups and subnetworks (Fig. 1c). Conversely, AI/ML models are trained to convergence and rely on precise and static sets of synaptic weights.

## Neurodynamical Computing as Oscillatory Articulation

Freeman (2000) considered the first building block of neurodynamics for a local neuron group to be collective activity patterns that reliably return to a stable nonzero fixed-point following an input or perturbation [139]. Nonzero fixed-point dynamics are robustly associated with the 1–2% sparse intrinsic connectivity and 80:20 excitatory/inhibitory neuron proportions that typify local circuit architectures of mammalian cortex and hippocampus. Depending on input levels, neuromodulation, or other state-dependent factors, this circuit structure supports additional population dynamics that may serve to partition constituent neurons’ spiking according to cycles in the collective activity, e.g., transient negative-feedback waves and stable limit-cycle oscillations, which Freeman labeled as the second and third building blocks [139]. Neural oscillations are measured extracellularly as the net synaptic loop currents in the local volume [139, 140] and the structure of prevalent frequency bands is highly conserved across mammals including humans [141–143]. Neuronal spike phase is measured as the relative alignment of spikes to oscillatory cycles and effectively constitutes a distinct spatiotemporal dimension of neural interaction that naturally supports sequence learning, generation, and chunking in biological [144–152] and artificial [153–156] systems. Because various lines of evidence indicate that neuronal spike phase and collective oscillations may be causally bidirectional [157–162], the discrete neural states organized by oscillatory cycles are candidate computational states.

Despite all the complexity of brains that causally links phenomena across levels, we posited above that there must be a base layer below which the net causal effects on functional states at that level and above become negligible. Placing this dividing line above the level of neurons entails that myriad biological details of dendrites, somatic integration, ion channels, cell metabolism, quantum states, etc., ideally do not influence the processing of computational states. As a consequence, we focus our search for the fundamental unit of cognitive computation on dynamical flows of energy over neural tissue. These flows include massive internal feedback, which is neglected by AI/ML methods for reasons including (ironically) incompatibility with error backpropagation. While the cell assembly has been incorporated into the everyday language of neuroscience, its intrinsically dynamical conception—by Karl Lashley and Donald Hebb [163]—has been diluted so as to support rigidly neurocentric notions like population codes and memory engrams that elide temporality by simply identifying cognitive states with subsets of coactive neurons [59, 164–166]. Instead, we should consider how a neural substrate might support the spatiotemporal waves and vortices that arise in the dissipative energetics of extended and embodied living systems. Additional theoretical research and modeling are needed to systematically analyze the spatiotemporal nonlinearities and constraints (e.g., [167, 168]) imposed by the ignition of reentrant sequence flows as units of computation.

Neurodynamical computing may thus undergird the cognitive flexibility critical to adaptively responding to disruptions or alterations of an organism’s situation, i.e., the frame problem. This flexibility necessarily trades off with the stability of active states [169, 170]; i.e., stable relaxation into the energy well of an attractor must be periodically interrupted to ensure responsivity to new inputs. For example, mice exhibit strong cortical-hippocampal oscillatory entrainment to respiration [114, 171, 172], which may serve to optimize odor sampling by rhythmically phase-locking sniffing behavior to the most responsive (i.e., unstable) neurodynamical states [98, 139, 173]. In rats, the famous theta rhythm (5–12 Hz) was revealed by its clear association, from early in the modern neuroscience era, with voluntary movement and complex ethological behaviors [174, 175]. More recent research has extended this association to show that theta controls the speed and initiation of locomotion behaviors [176–179]. Moreover, the practice of analyzing hippocampal data aligned to theta cycles has driven subsequent decades of neuroscience research relating oscillatory phenomena to lifelong learning, episodic memory, and spatial navigation in rodents and humans [149, 180–183]. For instance, a place-cell remapping study in which global context cues (salient light patterns) were abruptly changed demonstrated rhythmic “theta flickering” [184, 185] in which each theta cycle would alternately express one or another spatial map until settling on the new map. Theta flickering, exposed by an unnaturally abrupt change in an animal’s physical context, suggests that the hippocampal cognitive map is completely torn down and reconstructed every single theta cycle. This continual rebuilding of active context-dependent states in hippocampal memory networks hints at the computational efficiency and power of sculpting and refining attractor landscapes over a lifetime. These deep behavioral and cognitive links to slow, ongoing neural oscillations—alternating between stability and instability—may reflect fundamental mechanisms of brain–body coordination [143, 172, 186].

Understanding how oscillations, behaviors, or other factors govern neurodynamical computing will require explicating the high-dimensional chaos that drives state transitions to the next attractor (or phase transitions to a new chart or landscape) [98, 187–192]. Crucially, several lines of evidence converge to emphasize the neurodynamical and control-theoretic complexity of systems that intermix positive and negative feedback. First, models of recurrent feedback circuits—like those typical in the brain—can be made functionally equivalent to a feedforward process by learning network states that act as linear filters for subsequent states in a sequence [193]. Second, chaotic attractors form at dynamical discontinuities, or cusps, corresponding to interfaces between positive and negative feedback processes [194]; for example, demonstrations of metastable transitions between inferred cortical states during taste processing [195–197] could, speculatively, reflect traversals across the discrete gustatory wings of a high-dimensional chaotic attractor. Third, singularity-theoretic modeling of mixed-feedback control in neuronal bursting and central pattern generator circuits has demonstrated that small perturbations around these *winged cusps* can evoke persistent bifurcations in dynamics [81, 198, 199], akin to a digital switch. We theorize that the ascending/descending streams of perceptual control could converge on this type of neurodynamical computing interface.

## Absorbed Coping as Internalized Entropy Control

Collectively, the above findings suggest neurodynamical articulatory mechanisms for producing itinerant trajectories of discrete computational states. Moreover, the philosopher Hubert Dreyfus (2007) suggested that the accumulation of articulations—via the adaptive, continual refinement and rebuilding of attractor landscapes through experience—may solve the frame problem for intelligent systems [13]. By identifying neurocognitive states, including memory states, with dynamically constructed states, we see that many biological complexities dissolve; but how can an intelligent organism or artificial agent use these principles to construct meaning? A recent study reported neural recordings from the brains of rats as they happily socialized with and learned to play hide-and-seek with human experimenters [200]. Neuroscience crucially requires new, innovative experimental paradigms like this that respect the autonomy, agency, and ethological behavior of the animals we study. Dreyfus (2007) further wrote that “[w]e don’t experience brute facts and, even if we did, no value predicate could do the job of giving them situational significance” [13]. Which is to say: significance, meaning, and relevance need to be built into our experiments and models from the beginning, and then refined with experience. Thus, taking an embodiment-first approach to cognition and intelligence is also, and not incidentally, a meaning-first approach. Many arguments about the possibility of neurosymbolic systems reflect questions about how to give these systems access to intentionality and meaning. A major implication of this perspective article is that we see no easy way around this problem.

Meaning comes from interacting over time with the world and learning about how those interactions affect your body and how they make you feel. In biology, organisms have visceral, affective, and interoceptive homeostasis built in, beginning with early development and undergoing continual refinement throughout life. That refinement occurs through learning and our experience as “absorbed copers” [13] trying to get through the problems of the day. In contrast, supervised training of static, disjointed datasets by iteratively propagating errors through densely connected neural nets is a recipe for the elimination of meaning. Instead, it is critical to rethink our science of intelligence as driven by integrative advances in AI, computational neuroscience, embodied cognition, and autonomous robotics. The agents we study must learn to perceive and cope with being in the world as we do. 
